# The Climate of the South of Devon, and Its Influence on Health, &c. &c.

**Published:** 1842-10-01

**Authors:** 


### The Climate of the South of Devon, and its Influence on Health, &c. &c. By *Thomas Shapter*, M.D. 8vo. pp. 258. Churchill, London, 1842.

Devonshire is running a sharp race, in the sanitary course, with Italy—Torquay with Nice—Hastings with Pisa—and Undercliff with the Eternal City itself! The contrast between the bright skies of Italy and foggy atmosphere of England has led more people over the Alps, in search of health, than an accurate investigation of the bills of mortality in both countries would have done. That a climate which consigns a twenty-fifth part of its inhabitants annually to the grave, should be preferred to one where only a fortieth or fiftieth part pay the penalty of Nature, is strange. There is no doubt, however, that certain seasons of a climate generally unfavourable to longevity, may prove salutary in certain complaints and constitutions. Thus a *winter* in Italy may mitigate a pulmonary complaint contracted in England; but it is only in the early stages of such maladies that the mildness of Roman air can at all prove serviceable. If this truth had been well understood, the pyramid of Caius Cestius would not now sweep its funereal shade over the tombs of so many sons and daughters of Albion—who left their native homes only to mingle their ashes with those of the Cæsars, the Antonines, and the Neros, in the animalized soil of Rome!

The work under review is divided into two parts—but the second part being geological and hydrological, it is to the first part only that we can refer in this article.

"The more striking characteristic of the climate of Devon generally, is that of being warm and moist: though this is partly owing to its latitude, yet much is due to its position as regards the ocean, forming, as it does, a portion of a large promontory, or imperfect peninsula, projecting westward into the Atlantic, so that nearly one half of its circumference is sea coast. In this latter circumstance alone conditions exist, which not only ameliorate and soften its general character, but likewise tend to produce an equability of temperature, which is not common to larger breadths of land." 2.

The surface of Devon presents a pleasing succession of undulating hills and luxuriant vales, in the highest state of cultivation, and richly wooded throughout. The mean annual temperature is 52°, 29—about one degree higher than that of London—or probably more. Equability of temperature is a striking character of Devon—the difference between the warmest and coldest of ten years being only four degrees. In the seasons, the greatest change of temperature takes place between the spring and summer, amounting to more than 12½ degrees—the least variation is between winter and spring—being less than 8 degrees. This is very different from London and other places in England.

The barometrical phenomena are not materially different from those observed in London, except in shewing a slighter amount of higher measurement, together with a tendency to less variation.

The moisture of Devonshire is proverbial, and this character is fully sustained by Daniel's hygrometer. The winter season is the most damp—the autumn is next, and the spring and summer are comparatively dry. The dampest month is November. "In general terms it may be stated that, from March to September, the climate is dry—and during the remainder of the year humid."

The mean annual fall of rain in the southern part of Devon is about 32 inches—seven inches more than in London. The largest quantity falls in the autumn and winter—the least in the spring. The number of wet days is not so great in Devonshire as in London. Frost, during the winter and spring, is not unfrequent, but is seldom of any duration. Except on the high grounds, snow seldom continues on the ground more than a few days. The great prevailing winds are from the west and north-west. They are much more frequent than in London. The following is our author's summary of the climate of the South of Devon.

"It may be seen from the previous sketch that the chief characteristic of the climate of this district is that of being warm, soft, mild, equable, calm, and free from storms; though subject to a large share of rain, yet it seldom occurs that a whole day is so unceasingly wet, as not to afford some hours, whether early or late, sufficiently fine for outdoor exercise. During the winter season the temperature rarely maintains for any length of time a degree so low, as to render the climate particularly inclement; frost seldom occurring, and rarely of long continuance. The air is usually damp; but from the general prevalence of warm westerly winds, the moisture which it contains is not cold and chilling. A general impression prevails that this, which may be styled the muggy weather of Devon, is unwholesome, such, however, is not the case, as will be seen in the sequel; in fact the accompanying temperature takes from it the usual injuriousness of such a condition. The character of the spring during the early part does not materially differ from the winter, excepting that the air is less damp, and the days less rainy. Towards the close of this season north-easterly winds somewhat prevail; these should be particularly guarded against, especially by the delicate, as from their piercing and cold nature, they are apt, by repressing the cutaneous functions, to cause internal inflammation. The summer is rarely very hot, and though showers are frequent, yet it may be considered a dry season. The winds which blow, for the most part from the north-west, are cooling and refreshing. The evenings and nights, however, are sometimes cold and damp, and, therefore, exposure at these times, with only summer clothing, should be avoided. The autumn is warm, and inclined to be damp and rainy; it is peculiarly the season for the Devonshire drizzle, which is a rain so light as to deposit itself as a thick dew, attended by a gray-clouded sky: the winds during this season are chiefly from the west. By way of marking the general mildness of the climate, it may be mentioned that many of the tender and delicate exotics flourish in the open air, and are not destroyed by exposure during the winter season. During this period also, it is not unfrequent to see the hedges studded with many of the native flowers." 39.

We must pass over a great many pages of the work treating of the most prevalent diseases in Devonshire; because few people go thither to contract diseases, but to get rid of them. We must make an exception, however, in the subject of

### Diseases of the Lungs.

Seventeen per cent. of the whole catalogue—or about a sixth of the whole of the diseases of Devonshire, comes under the head of pulmonary complaints. Of these, a large proportion consists of simple inflammation of the bronchial tubes—the symptoms of which we need not describe. Chronic catarrh is very common among the lower orders. Bronchitis prevails in the spring and winter.

"Consumption forms a large proportion of the diseases of the chest in this district; it occurs in all its various forms, though, most usually, the cases are protracted and lingering.

Amongst the cases of phthisis, a few were instances of pure phthisis laryngea. In these no very marked symptoms of any affection of the lungs were observable; on examination after death, however, they were invariably found much impacted by tubercular deposit. A few cases have also occurred of phthisis, complicated with syphilis. In these the alterations of disease were quite remarkable: at times the chest complaint would appear to be entirely suppressed, while syphilis, in its various forms of periostitis and ulceration, was rapidly progressing; then, on these subsiding, the more fatal affection, would develope itself; eventually they died of consumption in which both the perspiration and expectoration were peculiarly offensive. Three cases of mental delusion, in connexion with consumption, and after free salivation by mercury, have occurred to my observation, with so much singular coincidence, that I am induced not only to refer to them here, but to style them phthisis, complicated with mercurial irritation.

In each the patients presented the usual character of the incipient stage of phthisis; but superadded to this was an impression that their whole system was impregnated with mercury, which in two cases had been taken for syphilitic affection, and in the third for an accidental attack of swelled testicle. So strongly rooted was this impression that they maintained they smelled it in their perspirations, tasted it in their saliva, were convinced it was in their secretions, and that to this, and this only, was attributable the unpleasantness of the symptoms they were labouring under. This state of things in each occurred until the symptoms of phthisis became fully developed, which was usually sudden; then the delusion subsided, and the patient went through the ordinary course of a very rapid decline." 92.

But we must now pass on to notice some particular localities.

### EXETER.

This city occupies the flat summit and the declivities of a hill, on the eastern bank of the river Exe, one hundred and fifty feet above the level of the sea. It thus presents great facilities for cleanliness and ventilation; but we fancy that few invalids will pitch their tents in the Devon Metropolis, who can extend their steps to

### TORQUAY.

This has, for several years, been celebrated as a suitable winter residence for pulmonary invalids.

"It is situated in a cove at the north-west angle of Torbay. This cove is surrounded by three hills, 'nearly equal in elevation, (180 to 200 feet) and similar in general features. Between them run two tortuous valleys, one towards the east, the other towards the north. It is on the shores of the cove, along the slopes of the hills, and in the gorges of the valleys, that the town is built,' facing the south-west, and sheltered from the north and east. It presents the appearance of a number of rising terraces, which receiving the direct rays of the sun, unchilled by the colder winds, form safe and pleasant walks for the invalid. The scenery from these terraces is peculiarly beautiful; immediately beneath is expanded the bay, like a small inland sea, on every side bounded to the view by wooded heights.

The climate of Torquay is generally understood to be amongst the warmest, and most genial upon the coast, but in the absence of sufficient information, its accurate position in this respect cannot be stated. Many observations on its temperature, &c. have certainly been registered; more especially by Mr. Blewitt, Drs. Coldstream and Barry, but at such irregular times, and for so short a period at each time, that it is quite out of the question deducing from them anything like satisfactory averages. From these, however, it appears that its mean annual temperature is rather more than fifty one degrees and a half (51°.65), nearly half a degree warmer than Exeter; and the mean winter temperature, above forty-four degrees (44°.25) which is more than three degrees warmer. The mean difference between the temperature of successive days in the cold season, may be stated at 2°.7; its daily range appears also to be small. The general mildness of this place may be somewhat appreciated by the statement of the lowest degree of cold experienced in the generally severe winter of 1837-8, being only 21°, while in Exeter it was 17°, Bristol 8°, Kensington 0, and at Sandhurst 8° below zero. Dr. Coldstream, during a winter residence at Torquay, (1832-3,) made a series of observations upon the temperature of his bed-room, at 7 A.M., in which no fire was kept; they are extremely interesting, and would be useful if more generally made; the temperature of the interior of houses being of the greatest importance to the invalid. From these it appears that within-doors the air was nearly two degrees (1°.80) warmer during the winter months, than the mean of the external atmosphere. This relative difference of temperature is much influenced, as might be expected, by the prevalence of wind, for we find that, in the comparatively calm months of December and January it is considerable, (2.54,) while in March it scarcely exceeds half a degree (0.58.) The air of Torquay is generally drier, and more free from fog than is common to the Devonshire climate; it is also said to be less subject to rain. In 1838 there fell only 35.1 inches, while in Exeter 38.9 were deposited, at Plymouth 40.6, and at Plympton 43.7. Though this is merely the statement of one year, and as such cannot be conclusive, yet it is to a certain extent confirmatory of an impression generally entertained." 143.

The town is chiefly supplied with water from a distance through iron pipes, and is clear and sparkling, with plenty of lime. Hot and cold baths are of easy access; but the accommodations for sea-bathing are not very good.

"Torquay is peculiarly suitable, during the winter, to persons labouring under chest complaints generally. Those far advanced in diseases of the lungs pass their time more easily to themselves, and freer from the harassing effects of cough and febrile irritation; while those in the early threatening of disease, may not unfrequently date a permanent re-establishment of health, to a residence in its mild climate.

### Babbicombe.

This small place, within a short drive to the eastward of Torquay, affords a climate directly its opposite, being open to the north and north-east. Though bleak and cheerless in winter, it forms in summer a most delightful residence; it is situated upon the slope of a steep hill, whose massive rocks, and thickly-planted grounds, constitute a scene as romantic as it is picturesque. This cove is the property of a few persons, who chiefly inhabit it themselves, and consequently affords but little accommodation to strangers. Between Babbicombe and Torbay there is a bone cavern, well worthy the attention of the curious." 145.

### Teignmouth.

This place is 15 miles from Exeter, at the mouth of the river Teign, partly on a flat, partly on the sides of high hills, which shelter it on the north. East Teignmouth faces the sea, and is exposed—West Teignmouth is more protected. The beach is open, and there the relaxing heat of summer is much modified. The climate partakes of the general equability of the district—the average temperature being six degrees higher than that of London in winter, and five degrees lower in summer.

"The sea-bathing is good, and available at all times of the tide; the sands are perhaps better than on any beach upon the coast. There are hot, cold, vapour, and medicated baths, which are conveniently situated, and well appointed. As a place of sojourn for the invalid, Teignmouth can be recommended from the beginning of June to the end of October: it is not so favourable during the remainder of the year, and in the spring is decidedly ill adapted to persons with weakly constitutions." 148.

Take it altogether, Teignmouth forms a pleasant place of residence, and is remarkable for many instances of longevity.

### Dawlish is another small and pleasantly situated watering place, about 12 miles from Exeter, in a valley opening to the sea, and protected from the winds. It presents an air of cheerful seclusion and quiet which is very inviting. Although for the most part Dawlish is inhabited by permanent residents, it contains good accommodations for occasional visitants. The sea-bathing here is very good, the sands forming a pleasant footing, and the adjoining cliffs affording adequate shelter from the colder winds. The walks and drives in the neighbourhood are agreeable and accessible. The climate is considerably warmer than that of Exeter, and perhaps of Torquay.

"During the autumn and winter months there can be no place upon this coast better adapted as a residence for those suffering under pulmonary disease, so entirely is it protected from the prevailing winds of these seasons: this is more particularly the case with the little hamlet of Dawlish Water, a spot which has struck me as singularly mild. During the spring months, however, I should be inclined to regard Dawlish as not a very favorable residence, for patients of the above description, chiefly on account of the east winds which then prevail, and to which, from its aspect, it is peculiarly exposed." 150.

### Exmouth is situated about nine miles from Exeter, at the mouth of the river Exe, which extends here to a width of three miles. A portion of the town is built on low ground, while the remainder occupies the summit of a hill facing the sea. The exhalations from the mud are at times exceedingly offensive to the lower town.

"Notwithstanding the south-westerly aspect of Exmouth, it offers a more bracing climate than any of the watering-places here described. It may almost be said to be unsheltered by any immediate neighbouring ground, though to a certain extent the Woodbury Hills protect it from the north." 153.

"From its aspect and open situation Exmouth presents no great objections as a residence, at any season of the year. It will be found particularly serviceable as a resort for weakly children, and those of a scrofulous constitution, and where change is required in the debility consequent upon attacks of fever, or during convalescence after other diseases: no place upon the coast could be better chosen for these purposes. I should also strongly recommend it in irritable indigestions, catarrhal affections, and more especially in the dry asthma, which experience has often shown to be greatly benefited by it. Its exposed situation, I cannot but think, renders it injurious in the severer affections of the chest, and particularly in cases where there is a tendency to hæmorrhage or inflammation of the lungs, as also where consumption has been fully developed. To rheumatic complaints it appears particularly inapplicable—those liable to such affections invariably suffer. Female derangements are benefited by a residence in the higher part of the town, but in the lower there is too great a tendency to produce relaxation." 155.

### Sidmouth.

This is the last place mentioned by our author. It is a well-known watering-place, situated about 15 miles to the east of Exeter. It is built on a diluvial deposit of gravel overlying red sandstone. The surrounding hills are about 500 feet above the level of the sea, and shelter the town sufficiently. It is only exposed to the south, which is the sea view. The walks and drives are circumscribed by the steep highlands in the immediate vicinity of the town; but there are pleasant and sheltered walks in the valley.

"Both from its size and climate this place offers an agreeable residence to persons who have lived long in the warmer latitudes; it is well adapted for those labouring under affections of the liver; and during the autumn, winter, and spring seasons for the consumptive invalid; indeed, I am inclined to think that during the spring months it is the best place upon the coast for those liable to pulmonary complaints generally; during the summer it cannot be recommended, being then too hot and relaxing." 152.

We have not space to dwell on the long chapter on Geology. Devon, like many other places, presents granite, sandstones of all colours—trap—limestone—grauwacke, &c. &c.—and what is better than all, clouted cream and plenty of cider. We must also pass over the concluding chapter on "Vital Statistics," to which justice could not be done in this article. The work is very creditable to the industry and ability of the author.

